# Hepatitis C virus core or NS3/4A protein expression preconditions hepatocytes against oxidative stress and endoplasmic reticulum stress

**DOI:** 10.1080/13510002.2019.1596431

**Published:** 2019-03-25

**Authors:** W. Alfredo Ríos-Ocampo, Toos Daemen, Manon Buist-Homan, Klaas Nico Faber, María-Cristina Navas, Han Moshage

**Affiliations:** a Department of Gastroenterology and Hepatology, University of Groningen, University Medical Center Groningen, Groningen, Netherlands; b Department Medical Microbiology, University of Groningen, University Medical Center Groningen, Groningen, Netherlands; c Grupo Gastrohepatología, Facultad de Medicina, Universidad de Antioquia, Medellin, Colombia; d Department of Laboratory Medicine, University of Groningen, University Medical Center Groningen, Groningen, Netherlands

**Keywords:** Hepatitis C virus, cellular stress, oxidative stress, unfolded protein response, ER stress, apoptosis, Core, nS3/4A, Transient expression

## Abstract

**Objectives:** The occurrence of oxidative stress and endoplasmic reticulum (ER) stress in hepatitis C virus (HCV) infection has been demonstrated and play an important role in liver injury. During viral infection, hepatocytes must handle not only the replication of the virus, but also inflammatory signals generating oxidative stress and damage. Although several mechanisms exist to overcome cellular stress, little attention has been given to the adaptive response of hepatocytes during exposure to multiple noxious triggers.

**Methods:** In the present study, Huh-7 cells and hepatocytes expressing HCV Core or NS3/4A proteins, both inducers of oxidative and ER stress, were additionally challenged with the superoxide anion generator menadione to mimic external oxidative stress. The production of reactive oxygen species (ROS) as well as the response to oxidative stress and ER stress were investigated.

**Results:** We demonstrate that hepatocytes diminish oxidative stress through a reduction in ROS production, ER-stress markers (*HSPA5* [GRP78], *sXBP1*) and apoptosis (caspase-3 activity) despite external oxidative stress. Interestingly, the level of the autophagy substrate protein p62 was downregulated together with HCV Core degradation, suggesting that hepatocytes can overcome excess oxidative stress through autophagic degradation of one of the stressors, thereby increasing cell survival.

**Duscussion:** In conclusion, hepatocytes exposed to direct and indirect oxidative stress inducers are able to cope with cellular stress associated with viral hepatitis and thus promote cell survival.

## Introduction

Hepatitis C Virus (HCV) infection is a major infectious disease characterized by high morbidity and mortality. According to the World Health Organization (WHO), 71 million people have chronic HCV infection causing around 400,000 deaths each year worldwide [1]. Acute and chronic hepatitis caused by HCV can vary in severity and outcome although 60–85% of all cases progress to chronic infection [1]. The treatment of chronic HCV infection has been revolutionized with the introduction of direct-acting antivirals (DAA) and more than 95% of patients who complete DAA treatment eliminate the virus [2]. Despite these advances in therapeutic approaches, HCV is still an important global public health problem and many unanswered questions about HCV pathogenesis and biology remain.

HCV is an enveloped virus belonging to the *Flaviviridae* family. The viral genome is a positive-sense single-stranded RNA (+ssRNA), which encodes a polyprotein of around 3100 amino acids [3]. During viral replication, the polyprotein is co- and post-translationally cleaved into 4 structural (Core, E1, E2 and p7) and 6 nonstructural proteins (NS2, NS3, NS4A, NS4B, NS5A and NS5B) by host and viral proteases [4]. An additional protein, the F protein, may be the product of an alternative reading frame in the Core encoding sequence [5].

It has been demonstrated that HCV replication and expression of viral proteins induce cellular stress that may play an important role in the pathogenesis of liver injury and liver fibrogenesis [6,7]. The altered cellular homeostasis due to the infection can result in increased oxidative stress and/or endoplasmic reticulum (ER) stress. This may lead to an adaptive response to maintain or restore homeostasis and prevent cell death, but this has not been investigated in HCV-infected cells [8].

Increased oxidative stress has been observed in liver biopsies from patients with chronic HCV infection and the generation of reactive oxygen species (ROS) was significantly higher in patients infected with HCV compared to other liver diseases [9,10]. In addition, consequences of oxidative stress like free radical-mediated lipid peroxidation, steatosis and increased levels of pro-oxidant markers were also increased during chronic HCV [11].

HCV Core, NS3, NS4A and NS5A proteins can all induce oxidative stress directly, although Core appears to be the strongest inducer [12–15]. HCV Core (21 kDa) is a highly conserved protein and is the subunit of the viral capsid [16]. Core triggers ROS generation via multiple mechanisms such as induction of nicotinamide adenine dinucleotide phosphate oxidases 1 and 4 (NOX1, NOX4) and cyclo-oxygenase 2 (COX2) [17]. NS3 is a 67 kDa protein. Its N-terminal region has serine protease activity and its C-terminal region has an NTPase/helicase function. The enzymatic activity of NS3 requires the presence of NS4A as a cofactor [18].

HCV protein synthesis also induces ER stress in the host cell, as shown for Core and NS3/4A [19–22]. In response to ER stress, mammalian cells activate the Unfolded Protein Response (UPR). The UPR is an adaptive mechanism that reduces stress by enhancing protein folding, decreasing protein load at the ER and promoting the expansion and rearrangement of the ER membrane. The UPR is composed of three classes of ER-stress sensors: Protein kinase R (PKR)-like Endoplasmic Reticulum Kinase (PERK), Activating Transcription Factor 6 (ATF6) and Inositol-Requiring protein 1 (IRE1 or the human homologue Endoplasmic reticulum to nucleus signaling 1) [23]. Glucose-Regulated Protein 78 (GRP78, also known as immunoglobulin heavy chain-binding protein [BiP] encoded by the *HSPA5* gene) plays an important role as inducible chaperone in the UPR. Since HCV infection results in accumulation of HCV proteins in the ER, GRP78 can bind to unfolded viral proteins, triggering the activation of PERK, ATF6 and IRE1 [24,25]. Inability to resolve ER stress can lead to cell death by apoptosis [26].

During chronic HCV infection, the combination of oxidative stress and ER stress induced by viral protein synthesis poses a severe threat to the hepatocyte. The aim of this study was to investigate whether hepatocytes can resist the effects of direct and indirect oxidative stress e.g. by activating an antioxidant response and/or UPR. To answer this question, we decided to use transfected primary rat hepatocytes and human hepatoma cells expressing HCV Core or NS3/4A protein, exposed to external oxidative stress induced by the superoxide anion donor menadione (2-Methyl-1,4-Naphthoquinone) which has been extensively used to study redox biology of the cell [27–29]. In previous studies of our group, we demonstrated that menadione-induced apoptosis is mediated by superoxide anions and dependent on phosphorylation of c-Jun N-Terminal Kinases (JNK) and subsequent activation of caspase-9, -6 and -3 [30]. We found that hepatocytes expressing HCV proteins Core and NS3/4A are more resistant to external oxidative stress than non-infected hepatocytes.

## Methods

### Vectors and cloning

The mammalian expression vector pTracer^TM^-EF/V5-His (Invitrogen) was used as backbone for subcloning HCV Core and NS3/4A coding sequences. The expression of HCV Core and NS3/4A recombinant proteins was under the control of the human elongation factor 1α (hEF-1α) promotor. The expression of green fluorescent protein (GFP) under the control of the human cytomegalovirus immediate-early promotor was used to determine the transfection efficiency. Sets of primers were designed to amplify the HCV sequences from the full-length HCV JFH-1 replicon, genotype 2a (kind gift of Dr. Wakita from National Health Institute of Japan [Apath, strain reference APP1025]). Primer sequences are described in Supplementary Table 1. Fragments of 574 and 2,050 base pairs (bp) were amplified, corresponding to the sequences of Core and NS3/4A, respectively. The sequences were inserted into pTracer™-EF/V5-His, using the *EcoRI* and *Xba1* restriction sites. The pTracer™-EF/V5-His was used as a negative control (empty vector). Cloning and generation of plasmids were confirmed by sequencing (BaseClear, Leiden, The Netherlands).

### Isolation and transfection of rat primary hepatocytes

Primary hepatocytes were isolated from pathogen-free male Wistar rats (220–250 g; Harlan, The Netherlands) using a two-step collagenase perfusion method as described previously [31]. Trypan blue staining was used as viability test and only hepatocyte isolations with a viability above 85% were used. The animals were housed and treated following the guidelines of the local committee for care and use of laboratory animals from the University of Groningen. After isolation, 1.5 × 10^6^ hepatocytes were cultured in collagen-coated T25 flasks with William's E medium (Gibco, Cat N 32551020, United States of America, San Jose, California) (Supplementary Table 2 for detailed description of medium composition) supplemented with 50 μg/ml of gentamycin (BioWhittaker, Verviers) and 50 nmol/l of dexamethasone (Sigma) for 4 hours at 37°C and 5% CO_2_ to allow cells to attach. After the attachment period, cell cultures were 70% confluent and transfected with Lipofectamine™ 3000 transfection reagent (Invitrogen) and the expression vectors pTracerCore, pTracerNS3/4A and the empty vector (pTracer™-EF/V5-His), separately. A ratio 2 μl:1 μg (Lipofectamine 3000: plasmid vector) was used. The Lipofectamine 3000 and the plasmids were prepared in OPTI-MEM™ I (1X) reduced serum medium (Gibco) following the manufacturer’s instructions. Media was replaced 6 hours post-transfection (hpt) and hepatocytes were subsequently cultured in William's E medium supplemented with gentamycin (Gibco), and 1% penicillin-streptomycin (Gibco) for 24 hours. Transfection efficiency and cell toxicity were determined using flow cytometry and trypan blue exclusion staining, respectively. Experiments were conducted in duplicate wells and results are expressed as the average of three independent experiments.

### Cell sorting of rat primary hepatocytes

Rat primary hepatocytes were sorted by fluorescence-activated cell sorting (FACS) using a Beckman Coulter MoFlo XDP cell sorter. Fluorescent (GFP+) and non-fluorescent (GFP−) populations were harvested in FACS buffer (1X Hank´s Balanced Salt Solution [HBSS] Ca^2+^ Mg^2+^/10% fetal bovine serum [FBS]) 30 hpt. The negative population was used as control cells, since they had been exposed to DNA-lipofectamine complexes, but not transfected. To avoid cell damage we used a nozzle tip with a 100 µm diameter. The flow rate was kept at 6000–8000 events/sec. The yield was usually 1 × 10^6^–1.5 × 10^6^ cells.

### Transfection and treatment of hepatoma cell line Huh-7

Huh-7 cells were maintained in Dulbecco’s modified Eagle medium (1X) + GlutaMAX™- I (DMEM; Gibco, Cat N 10569010) (Supplementary Table 3 for detailed description of medium composition) supplemented with 10% FBS (Gibco) and 1% penicillin-streptomycin (Gibco) at 37°C and 5% CO_2_. Cells (3.0 × 10^5^) were seeded in 6-well plates and transfected after 24 hours. Confluence was 80%. Lipofectamine™ 3000 (Invitrogen) and the expression vectors pTracerCore, pTracerNS3/4A and the empty vector were used separately at a ratio of 4 μl:1 μg (Lipofectamine 3000: plasmid vector). The Lipofectamine 3000 and the plasmids were prepared in OPTI-MEM™ I (1X) reduced serum medium (Gibco) following the manufacturer’s instructions. Six hours after transfection the plasmid DNA-Lipofectamine complexes were removed and media was replaced. Transfection efficiency was determined using fluorescence microscopy and flow cytometry based on the expression of GFP 24 hours after media were replaced, which means 30 hpt. Additionally, cell toxicity after transfection was determined by trypan blue exclusion staining. Cells were treated 30 hpt with 50 µmol/l menadione, used as a donor of superoxide anions, to induce oxidative stress for 6 hours and viability was determined by trypan blue staining. As a control, Huh-7 cells were pre-treated 30 minutes before induction of oxidative stress with 5 mmol/l NAC (Sigma), an antioxidant, to suppress the menadione effect. Additionally, Huh-7 cells pre-treated with 5 μg/ml tunicamycin (Sigma) for 6 hours were used as a positive control for ER stress experiments. Experiments were conducted in duplicate wells and results are expressed as the average of five independent experiments.

### RNA isolation and RT-qPCR

Huh-7 cells and rat primary hepatocytes were harvested on ice and washed three times with ice-cold 1X HBSS. Total RNA was isolated with TRI-reagent (Sigma) according to the manufacturer’s instructions. Reverse transcription (RT) was performed using 2.5 µg of total RNA, 1X RT buffer (500 mmol/l Tris-HCl [pH 8.3]; 500 mmol/l KCl; 30 mmol/l MgCl_2_; 50 mmol/l DTT), 1 mmol/l deoxynucleotides triphosphate (dNTPs, Sigma), 10 ng/µl random nanomers (Sigma), 0.6 U/µl RNaseOUT™ (Invitrogen) and 4 U/µl M-MLV reverse transcriptase (Invitrogen) in a final volume of 50 µl. The cDNA synthesis program was 25°C/10 minutes, 37°C/60 minutes and 95°C/5 minutes. Complementary DNA (cDNA) was diluted 20X in nuclease-free water. Real-Time qPCR was carried out in a StepOnePlus™ (96-well) PCR System (Applied Biosystems, Thermofisher) using TaqMan probes, the sequences of the probes and set of primers are described in Supplementary Table 4. For qPCR, 2X reaction buffer (dNTPs, HotGoldStar DNA polymerase, 5 mmol/l MgCl_2_) (Eurogentec, Belgium, Seraing), 5 µmol/l fluorogenic probe and 50 µmol/l of sense and antisense primers (Invitrogen) were used. mRNA levels were normalized to the housekeeping gene 18S and further normalized to the mean expression level of the control group.

### Cellular oxidative stress and mitochondrial superoxide determination

The fluorogenic probe CellROX^®^ Deep Red Reagent (Invitrogen) was used to measure total cytoplasmic ROS according to the manufacturer’s instructions. After menadione treatment, 5 µmol/l of CellROX reagent was added to the cells. After incubation, media was removed and cells were washed three times with 1X HBSS Ca^2+^ Mg^2+^ (Gibco), harvested with 1X trypsin (Gibco) and analyzed by flow cytometry using a BD FACSVerse system and 635 nm laser. Mitochondrial production of superoxide anions was measured using MitoSOX™ Red reagent. 5 μmol/l MitoSOX™ reagent working solution was added to the cells. After 10 minutes, media was removed and cells were washed with 1X HBSS Ca^2+^ Mg^2+^ (Gibco) and harvested for flow cytometry analysis using a 488 nm laser. Five independent experiments were carried out and the results are expressed as average.

### Apoptosis and detection of caspase 3 activity

Apoptosis of transfected Huh-7 cells was detected using MitoProbe™ DilC1(5) combined with propidium-iodide (PI) (Thermo-Fisher Scientific, United States of America, Massachusetts) following the manufacturer’s instructions. After transfection, Huh-7 cells were harvested in FACS buffer and incubated with 50 nmol/l of DilC1(5) during 20 minutes at 37°C and 5% CO_2_. Cells were washed three times and pelleted in FACS buffer. Subsequently 1 µl of a 100 µg/ml PI solution was added and cells were incubated for 15 minutes at 37°C. Flow cytometry was performed using the BD FACSVerse system with 488 and 633 nm excitation lasers and analysis of apoptotic cells was plotted against reduction of DilC1(5) staining, indicating mitochondrial membrane potential disruption. Three independent experiments in duplicate were analyzed. A fluorometric assay was performed to determine caspase 3 activity in Huh-7 cells transfected and treated with menadione. Caspase 3 activity was measured as described previously [32]. Fluorescence was quantified in a spectrofluorometer at an excitation of 380 nm and emission of 430 nm. The arbitrary units of fluorescence (AUF) from three independent experiments were used to calculate the results.

### Immunofluorescence microscopy

Huh7 cells (9.0 × 10^4^) were grown on glass cover slips placed in 12-well plates. After 24 hours, attached cells were transfected according to the protocol described above. 24 hpt, media were removed and cover slips were carefully washed three times with 1X HBSS Ca^2+^ Mg^2+^ (Gibco). Then, cells were fixed using a 4% paraformaldehyde solution in 1X HBSS Ca^2+^ Mg^2+^ (Gibco) for 10 minutes at room temperature and washed 3 times with 1X HBSS-10% FBS solution. Permeabilization was performed by incubation of the samples for 10 minutes in 1X HBSS containing 0.1% Triton X-100 (Sigma). 1% Bovine serum albumin (BSA, Sigma) in 1X HBSS + 0.1% Tween 20 (Sigma) solution was used to block non-specific binding of the antibodies for 30 minutes. Monoclonal antibodies against HCV Core (Clone B12-F8, kindly provided by prof. Dr. Mondelli [33] and HCV NS3/4A (Clone 8 G2, (Abcam)) were used at a dilution of 1:1000 in 1% BSA/1X HBSS in a humidified chamber for 1 hour at room temperature. Samples were subsequently washed three times with 1% BSA in 1X HBSS solution. Finally, cells were incubated with goat anti-mouse Alexa Fluor® 568 in 1% BSA/1X HBSS for 1 hour at room temperature in the dark. Slides were evaluated using fluorescence microscopy and analyzed by Leica ALS AF Software (Leica).

### Western blot

Cell lysates were resolved on Mini-PROTEAN^®^ TGX Stain-Free™ Precast Gels (BioRad, UK, Oxford). Semi dry-blotting was performed using Trans-Blot Turbo Midi Nitrocellulose Membrane with Trans-Blot Turbo System Transfer (BioRad). Ponceau S 0.1% w/v (Sigma) staining was used to confirm protein transfer. The monoclonal antibodies human anti-HCV Core B12-F8, kindly provided by prof. Dr. Mondelli [33] and mouse anti-HCV NS3/4A (8 G-2; Abcam) were used at a dilution of 1:1000 and mouse anti-Glyceraldehyde 3-phosphate dehydrogenase (GAPDH) (Calbiochem) at a dilution of 1:10,000. Polyclonal rabbit anti-Microtubule Associated Protein 1 Light Chain 3 Beta (LC3B) (Cell Signaling) and anti-p62/SQSTM1 (Sequestosome 1) (Cell Signaling) were used at 1:1000 dilution. Secondary horseradish peroxidase (HRP)-conjugated antibodies were used. The blots were analyzed in a ChemiDoc XRS system (Bio-Rad). Protein band intensities were quantified by ImageLab software (BioRad).

### Statistical analysis

All experiments were performed using at least three different hepatocyte isolations for the rat primary hepatocytes and at least three independent experiments using Huh-7 cells. The average ± standard deviation (s.d.) were calculated for each experiment. The Graphpad Prism 5 software (GraphPad Software) was used for statistical analysis and comparisons were evaluated by unpaired, two-tailed *t*-test. For the group analysis two-tail ANOVA and Bonferroni post-test were performed. A *p* value of <0.05 was considered statistically significant.

## Results

### Reactive oxygen species production is attenuated in hepatocytes expressing HCV Core or NS3/4A proteins

**Figure 1.**
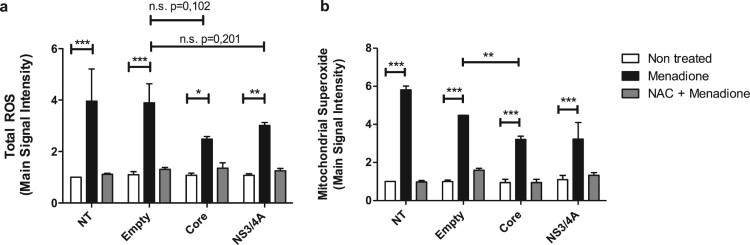
Reactive oxygen species production is attenuated in hepatocytes expressing HCV Core or NS3/4A proteins and under external oxidative stress induction. Total reactive oxygen species (a) and mitochondrial superoxide anion production (b) were detected using a set of fluorogenic probes (Cell ROX^®^ Deep Red Reagent and MitoSOX™ Red Reagent, respectively) in Huh-7 cells transiently transfected with the empty vector, pTracerCore or pTracerNS3/4A. Cells were treated with menadione (50 μM) 24 hours post-transfection for 6 hours. As a control and to inhibit the effect of menadione, cells were pre-treated 30 minutes prior to menadione treatment with the anti-oxidant NAC (5 mM). 2-way ANOVA was performed for group comparisons of the means with a Bonferroni post-test and in specific cases a *t* test was applied to compare the means between single comparisons; the asterisks represent the *p* value as: ***<0.001, **<0.003 and, *<0.05. (*p* value > 0.05). NT = No treated cells. n.s. = not significant. Experiments were conducted in duplicate wells and results are expressed as the average of three independent experiments.

Huh-7 cells were transiently transfected with HCV Core or NS3/4A expression vectors and transfection efficiency was approximately 60% ± 4.8 (Figure S1a and S1b). Cell viability after transfection was 95% ± 1.4, 92.5% ± 3.5, 94.5% ± 2.12 and 95% ± 1.4 for not transfected (NT), Empty, Core, and NS3/4A transfected cells respectively, which suggests that HCV Core and NS3/4A expression did not induce a cytotoxic effect (Figure S1c). The expression of Core and NS3/4A proteins was demonstrated by Western blot and immunofluorescence (IF) 30 hpt (hours post-transfection) (Figure S1d and S1e). The superoxide anion donor menadione significantly increased total ROS levels, which was blocked by the anti-oxidant NAC (N-Acetyl-L-cysteine) (Figure S2). Transfection of Huh-7 cells did not affect total ROS production (Figure 1(a): white bars). Although not statistically significant, menadione-induced total ROS production tended to be lower in Core and NS3/4A transfected Huh-7 cells compared to empty vector transfected and NT Huh-7 cells (Figure 1(a)). Since HCV Core and NS3/4A can translocate to the mitochondria, mitochondrial superoxide anion production was also evaluated (Figure 1(b)). As before, transfection did not affect mitochondrial superoxide production (Figure 1(b): white bars). Mitochondrial ROS production was significantly increased after menadione treatment. Interestingly, mitochondrial ROS production was significantly reduced in cells expressing HCV Core and additionally exposed to external oxidative stress (menadione treatment) compared to cells expressing the empty vector and treated with menadione, indicating less toxicity during double stress. A similar trend was observed in NS3/4A expressing cells treated with menadione, however, there was nosignificant difference when compared to Huh7 cells expressing the empty vector, probably because of the high variability between experiments (Figure 1(b)).

### mRNA levels of antioxidant enzymes are not affected by expression of viral proteins or exposure to oxidative stress

**Figure 2.**
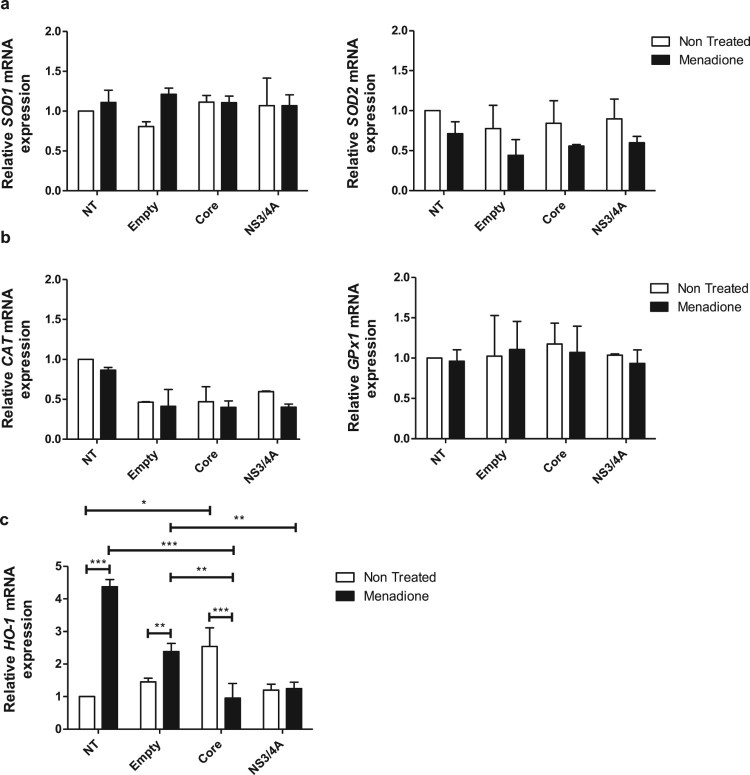
Antioxidant enzymes are not regulated by expression of viral proteins or exposure to oxidative stress in Huh-7 cells. The mRNA expression levels of the antioxidant SOD enzymes *SOD1* and *SOD2* (a), scavenging enzymes of H_2_O_2_, *CAT* and *GPx1* (b), and *HO-1* (c) were determined using qPCR in Huh-7 cells transiently expressing the empty vector, pTracerCore or pTracerNS3/4A with or without menadione treatment for 6 hours. The relative mRNA expression was normalized to the expression of 18S. 2-way ANOVA was performed for a group comparison of the means with a Bonferroni post-test and *t* test was applied to compare the means between single comparisons; the asterisks represent the *p* value as: ***<0.001, **<0.003 and, *<0.04. (*p* value > 0.05). NT = No treated cells.

In response to oxidative stress, cells can activate an enzymatic antioxidant response for ROS detoxification. Therefore, we investigated the expression of key anti-oxidant genes in Huh-7 cells transfected with viral proteins and exposed to menadione as oxidative stressor. The mRNA expression of both cytosolic copper and zinc-dependent superoxide dismutase (*SOD1*) and mitochondrial manganese-dependent superoxide dismutase (*SOD2*) did not change in response to expression of viral proteins and after menadione treatment (Figure 2(a)). Likewise, the mRNA expression of two additional important anti-oxidant genes, catalase (*CAT*) and glutathione peroxidase 1 (*GPx1*) were not changed by any of the interventions (Figure 2(b)). The heme oxygenase-1 (*HO-1)* mRNA expression (Figure 2(c)) was determined to analyze activation of the Nrf2/ARE (nuclear factor E2-related factor 2/antioxidant responsive element) pathway as well as an indirect marker of oxidative stress [34]. Expression of *HO-1* was significantly induced after menadione treatment in non-transfected cells. Transfection with empty vector had no significant effect on *HO-1* expression, indicating that transfection did not induce oxidative stress. In contrast, expression of Core alone did induce *HO-1* mRNA confirming its capacity to induce activation of the Nrf2/ARE pathway and confirming the pro-oxidative role from HCV Core [13]. Expression of NS3/4A did not induce *HO-1* expression (Figure 2(c)). Additionally, in Core-transfected cells exposed to menadione, *HO-1* mRNA expression was significantly reduced compared to menadione-exposed non-transfected cells or cells transfected with empty vector (Figure 2(c)). According to these results, the enzymatic antioxidant response, with the exception of HO-1, was not changed, at least not at the transcriptional level, by the expression of viral proteins or exposure to menadione.

### Core induces gene expression of heme-oxygenase-1 in rat primary hepatocytes.

**Figure 3.**
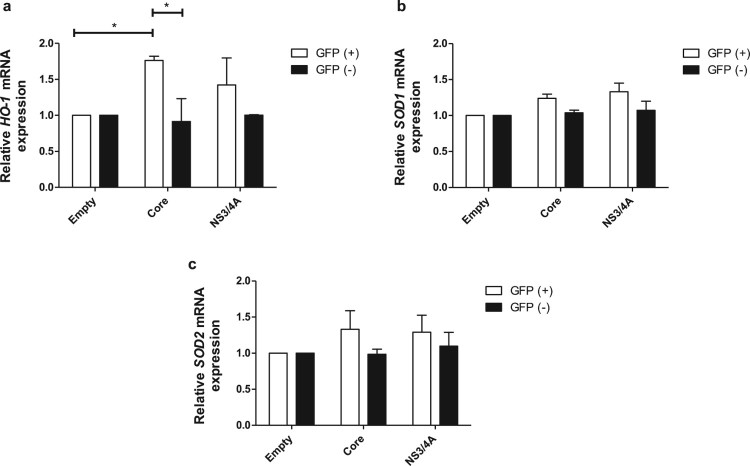
Core induces gene expression of heme-oxygenase-1 in rat primary hepatocytes. Hepatocytes were transfected with the empty vector, pTracerCore and pTracerNS3/4A separately. 24 hpt cells were harvested and sorted according to the expression of GFP. Transfected [GFP(+)] and not transfected [GFP(−)] cells were obtained. (a) The mRNA expression of *HO-1* was significantly increased in primary hepatocytes expressing HCV Core but not NS3/4A. The mRNA expression of antioxidant enzymes (b) *SOD1* and (c) *SOD2* was not changed in hepatocytes expressing HCV Core or NS3/4A. mRNA levels were quantified by qPCR. Relative expression was normalized to 18S. *t* test was performed to compare the means and the asterisks represent *p* value *<0.03. (*p* value > 0.05). NT = No treated cells.

To confirm the results obtained with transfected Huh-7 cells and to determine if HCV proteins are able to induce expression of antioxidant enzymes we repeated part of the experiments in primary rat hepatocytes. Rat primary hepatocytes were transiently transfected with the empty vector and pTracerCore or pTracerNS3/4A. Transfection efficiency was determined using flow cytometry of GFP positive cells; transfection efficiency was 9.5% ± 3.2 for the empty vector, 16% ± 4.2 for pTracerCore, and 10.5% ± 0.7 for pTracerNS3/4A (Figure S3a). The expression of Core and NS3/4A in primary rat hepatocytes was confirmed by Western blot (Figure S3b). After transfection, primary hepatocytes were sorted based on the expression of GFP. The expression of *HO-1* was significantly increased in hepatocytes expressing HCV Core, but not NS3/4A protein, confirming the pro-oxidant role of Core protein and the results in Huh-7 cells (Figure 3(a)). Expression levels of the antioxidant genes *SOD1* (Figure 3(b)) and *SOD2* (Figure 3(c)) were not affected by expression of Core or NS3/4A proteins, in line with the results obtained with Huh-7 cells.

### Hepatoma cells expressing core and NS3/4A are resistant to apoptotic cell death induced by oxidative stress

**Figure 4.**
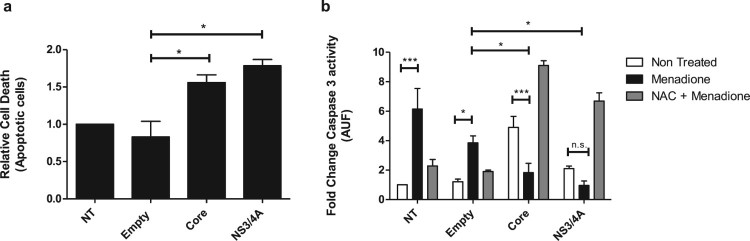
Hepatocytes expressing Core and NS3/4A are resistant to apoptotic cell death induced by oxidative stress. (a) Huh-7 cells were transfected with empty vector, pTracerCore and, pTracerNS3/4A. 24 hpt apoptotic cells were detected using DilC1(5) and propidium iodide (PI) and evaluated by flow cytometry according to manufacturer`s instructions. Expression of HCV Core and NS3/4A alone induces minor apoptosis. (b) Caspase 3 activity was determined in transfected Huh-7 cells treated with menadione (50 μM). Caspase 3 activity was significantly reduced in menadione-treated cells expressing HCV Core and NS3/4A compared to cells transfected with the empty vector. Treatment with the antioxidant NAC (5 mM) restored and even aggravated the apoptotic profile of HCV Core and NS3/4A. 2-way ANOVA was performed for group comparisons of the means with a Bonferroni post-test and in specific cases a *t* test was applied to compare the means between single comparisons; the asterisks represent *p* values: ***<0.001 and *<0.05. (*p* value > 0.05). NT = No treated cells.

To determine whether HCV Core and NS3/4A-expressing cells are protected against external oxidative stress-induced apoptotic cell death, we exposed Core or NS3/4A-expressing Huh-7 cells to menadione. Transfection alone (empty vector) did not affect survival of cells compared to non-transfected cells (Figure 4(a)). A slight increase in apoptosis was observed in Huh-7 cells expressing HCV Core and NS3/4A (Figure 4(a)). Menadione treatment significantly induced caspase 3 activity (Figure 4(b)). After external oxidative stress induction, apoptosis was significantly reduced in cells expressing Core and NS3/4A compared to cells transfected with the empty vector, suggesting an anti-apoptotic role of these proteins during oxidative stress induction (Figure 4(b)). To confirm our results, cells were also treated with the antioxidant NAC to suppress the effect of menadione. As shown in Figure 4(b) (gray bars), treatment with NAC restored the pro-apoptotic profile of Core and NS3/4A.

### ER-stress is reduced in hepatocytes expressing HCV NS3/4A after external oxidative stress induction

**Figure 5.**
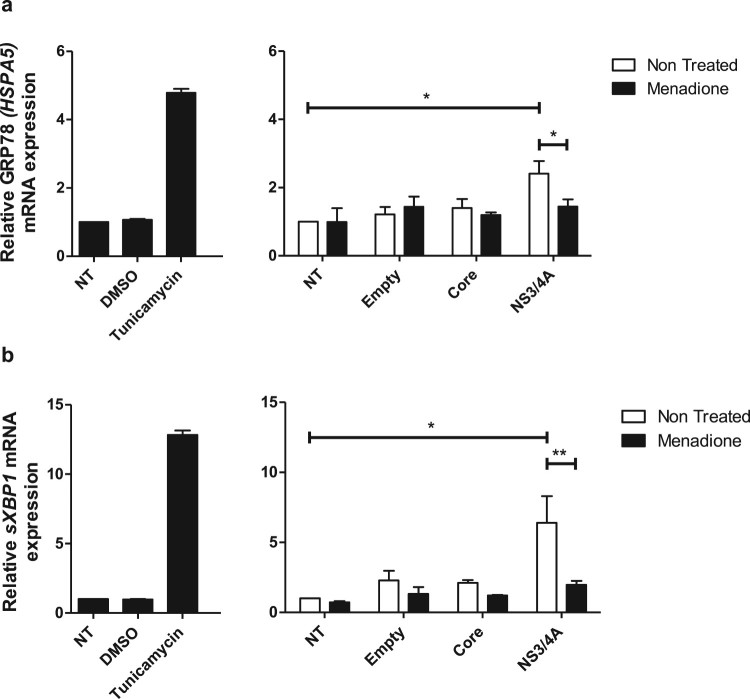
ER stress is reduced in hepatocytes expressing HCV NS3/4A proteins after external oxidative stress induction. ER stress was assessed by determining mRNA expression of the ER stress markers GRP78 (*HSPA5*) (a) and *sXBP1* (b). Tunicamycin was used as a positive control to induce ER stress. Transfection with NS3/4A, but not with empty vector or Core, induced ER stress comparable to the ER stress induced by tunicamycin (5 μg/ml). In our model (menadione treatment), NS3/4A-induced ER stress was significantly reduced (a and b). *t* test was performed to compare the means of mRNA expression and the asterisks represent the *p* value: ** < 0.01 and *<0.05. (*p* value > 0.05). NT = No treated cells. DMSO = Dimethyl sulfoxide.

Accumulation of viral proteins and RNA intermediates at the ER during HCV replication generate stress. Transient protein expression can also induce ER stress. To elucidate the ER stress profile in Huh-7 cells expressing Core and NS3/4A and after external oxidative stress induction, the mRNA levels of the ER stress markers GRP78 (*HSPA5*) and *sXBP1* (spliced X-box binding protein 1) were determined (Figure 5). Transfection with empty vector or Core did not affect the expression of GRP78 (*HSPA5*) (Figure 5(a)) or *sXBP1* (Figure 5(b)) in Huh-7 cells. In contrast, transfection of NS3/4A induced a statistically significant increase of GRP78 (*HSPA5*) and *sXBP1* mRNA levels, comparable to the induction observed with the ER stress inducer tunicamycin (Figure 5(a,b)). Interestingly, when cells were additionally treated with menadione (external oxidative stress induction), NS3/4A-induced ER stress was significantly reduced (Figure 5(a,b)).

### P62 may be involved in the reduction of oxidative stress via degradation of HCV Core protein

**Figure 6.**
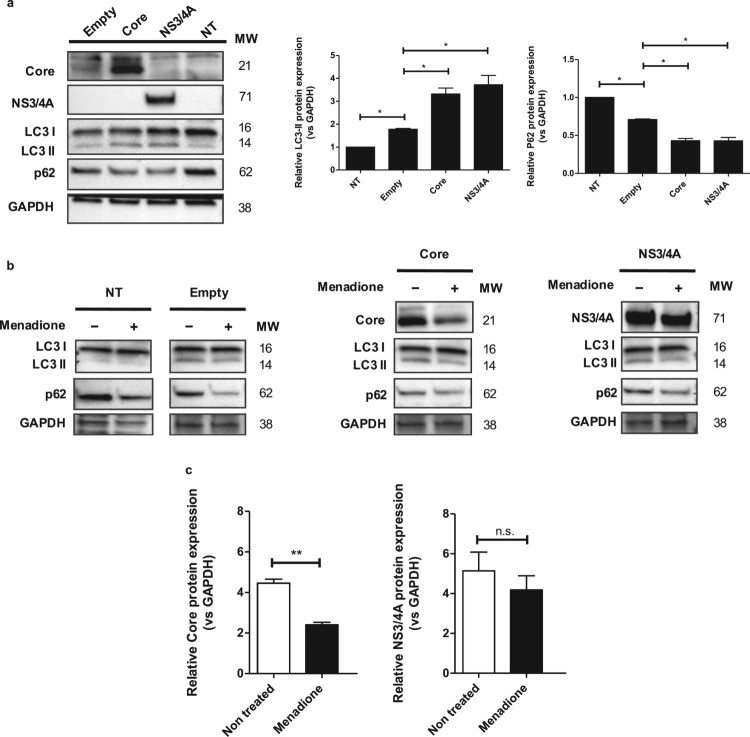
p62 may be involved in the reduction of oxidative stress via degradation of HCV Core protein. (a) Huh-7 cells were transfected with the empty vector, pTracerCore and pTracerNS3/4A. 24 hpt the expression of LC3-I/II, p62, Core, NS3/4A and GAPDH (loading control) were determined by Western blot. Protein expression of LC3-II was increased, whereas expression of p62 was significantly decreased in cells expressing HCV Core and NS3/4A indicating autophagy. (b) Treatment with menadione also decreased expression of p62, whereas protein levels of LC3-II remained stable. Cells expressing HCV Core and NS3/4A also displayed clearly reduced levels of p62 whereas levels of LC3-II remained stable. (c) After menadione treatment, the protein level of HCV Core and NS3/4A was quantified. HCV Core was significantly reduced while the protein level of NS3/4A remained stable. *t* test was performed to compare the means from the densitometry analysis and the asterisks represent *p* values: **<0.06 and *<0.02. (*p* value > 0.05). NT = No treated cells. MW = Molecular weight.

Autophagy is a survival mechanism after oxidative stress and ER stress [8,35]. To explore the hypothesis that autophagy may be involved in the Core/NS3/4A-mediated adaptation to menadione-induced oxidative stress and cell death, we investigated the modulation of autophagy proteins, such as LC3 and p62/SQSTM1 (ubiquitin-binding protein p62/Sequestosome-1), in our model. Core and NS3/4A expression resulted in significantly increased LC3-II (Microtubule-associated protein 1A/1B-light chain 3 (LC3)-phosphatidylethanolamine conjugate) levels and simultaneous degradation of p62/SQSTM1 (Figure 6(a)). This autophagy profile was similar to the profile observed in Huh-7 cells under starvation for 2 hours (Figure S4). Menadione treatment induced degradation of p62/SQSTM1 in non-transfected cells, Huh-7 cells transfected with empty vector and in Huh-7 cells transfected with Core and NS3/4A, whereas LC3-II levels did not change in response to menadione (Figure 6(b)). Interestingly, when cells were treated with menadione (external oxidative stress induction), HCV Core protein level was significantly decreased in response to menadione, while the level of NS3/4A protein remained stable (Figure 6(b,c)).

## Discussion

During HCV infection, hepatocytes are exposed to direct and indirect stressors. We hypothesized that cells infected with HCV virus, may resist to these stressors, conferring a survival advantage to the infected cells, thus sustaining the viral infection. Firm evidence for this adaptive response and its mechanism is lacking, mainly due to the lack of suitable model systems to investigate this adaptive response. In this study, hepatocytes expressing HCV viral proteins are subjected to an additional stressor to mimic different sources of damage reflecting the *in vivo* situation. We chose oxidative stress as the additional stressor because of the close association between HCV infection and oxidative stress observed in clinically relevant liver samples and animal models [9,36]. In our model, oxidative stress was generated by the superoxide anion donor menadione which has been extensively used before in redox studies [30]. It is extremely difficult to reproduce the HCV replication cycle *in vitro* in primary non-transformed hepatocytes, because of the rapid dedifferentiation, the species-specificity, the limited life-span of cultured primary hepatocytes and their resistance to transfection procedures [37,38]; nonetheless, some reports exist in the literature [39]. Therefore, we used Huh-7 hepatoma cells expressing the HCV proteins Core or NS3/4A to mimic the stress of viral protein synthesis. Yet, in some experiments we did use primary, non-transformed hepatocytes to validate our results. HCV Core and NS3/4A proteins were chosen since they are known to induce oxidative stress and ER stress respectively [12–14]. In addition, it has been described that these proteins are localized in membranous structures like mitochondria and ER and therefore they are relevant with respect to modulation of mitochondrial redox state and ER stress [19,40].

There are only a few reports that investigated adaptive mechanisms of HCV-infected liver cells [21,41]. Seo et al., reported that HCV Core expressing HepG2 and Huh-7.5 cells are more resistant to hydrogen peroxide (H_2_O_2_)-induced toxicity. H_2_O_2_ treatment increased the levels of protein p14 and induced the ubiquitin-dependent degradation of mouse double minute 2 (MDM2) protein with a subsequent reduction of MDM2-p53 interaction, accumulation of p53 and activation of p53-dependent apoptotic pathways. In this model, HCV Core decreased p14 expression, resulting in inactivation of the p14-MDM2-p53 pathway [41].

In our study, we demonstrate that HCV Core expression attenuates menadione-induced mitochondrial ROS production as well as Core and/or NS3/4A attenuates apoptotic cell death. Although Core expression did not lead to a significant reduction in total ROS production, there was clearly a trend towards reduced total ROS production in cells expressing Core or NS3/4A, probably due to the variability observed between experiments (*n* = 3). Furthermore, it has been described that selective depletion of only mitochondrial anti-oxidant status may provoke significant detrimental effects in hepatocytes [42]. A very interesting observation is that antioxidants restore the sensitivity of Core and NS3/4A expressing cells to undergo apoptosis, indicating that some level of ROS production is essential for the protective effect of Core and NS3/4A against oxidative stress. These observations correlate well with the observed expression pattern of HO-1 mRNA. We and others have previously shown that HO-1, and its products bilirubin and carbon monoxide (CO) have antioxidant and anti-apoptotic effects. In fact, we have shown that CO protects against menadione-induced hepatocyte apoptosis [43]. Our results are also in line with the phenomenon of preconditioning in ischemia-reperfusion injury, in which donor organs are exposed to a low level of oxidative stress, which protects against or attenuates subsequent major reperfusion injury. This phenomenon is, at least partially, mediated by HO-1 [44] and is in line with our results that expression of Core induces a low level of oxidative stress and *HO-1* expression. Many genes responsive to oxidative stress, including *HO-1*, are regulated by the transcription factor Nrf2 which binds to Antioxidant Response Elements (ARE) in the promotor sequences of antioxidant genes [45]. It has been shown that HCV infection can also activate Nrf2 and apparently Core and NS5A play an important role in this process [46,47]. However, we did not observe transcriptional regulation of other major anti-oxidant genes like *SOD1* and *SOD2*, *CAT* and *GPx1*, although we cannot rule out regulation at the post-transcriptional level.

Another mechanism by which HCV infection could interfere in cellular stress pathways is ER stress and the response to ER stress, the UPR system. ER stress is characterized by activation of the UPR via one or more of the signal transduction pathways PERK, ATF6 and IRE-1. The UPR serves to diminish ER stress [21]. HCV infection will lead to the accumulation of viral proteins in the ER and viral protein synthesis can lead to ER stress [48,49]. Consequences of ER stress and UPR are increased mRNA levels of GRP78 (*HSPA5*) that acts as an inducible chaperone in the UPR and sXBP1 activation [23]. We observed a clear activation of the UPR in response to ER stress in Huh-7 cells expressing NS3/4A, but not in Huh-7 cells expressing Core. This is in line with a previous studies showing that NS3/4A is able to induce ER stress [50–52]. Interestingly, when cells were exposed to external oxidative stress, the ER stress response and UPR activation in response to NS3/4A protein synthesis were reduced, indicating that despite the direct and indirect stressors in our model, hepatocytes attenuates not only apoptotic cell death but also ER stress.

Finally, we investigated another stress response, autophagy, in our model using direct and indirect stressors. Autophagy has been described as a critical survival mechanism against a variety of death stimuli, including oxidative stress [35]. Wang et al. reported that inhibition of autophagy in hepatocytes exposed to menadione-induced oxidative stress sensitizes cells to death from non-toxic concentrations of menadione and that autophagy-related pathways, like chaperone-mediated autophagy (CMA), plays an important role in this acquired resistance to oxidative stress [29]. The inhibition of CMA sensitized the cells to death since oxidized proteins, pro-oxidant proteins and damaged organelles can no longer be degraded [53]. In our experiments, expression of Core and NS3/4A resulted in significantly increased LC3-II levels and simultaneous degradation of p62. This autophagy profile was similar to that observed in Huh-7 cells under starvation for 2 hours, indicating a shift towards autophagy. Menadione treatment alone only induced degradation of p62, but LC3-II levels did not change, indicating that menadione alone did not induce a shift from apoptosis to autophagy. We hypothesize that the expression of Core and NS3/4A shifts the cells towards autophagy and that this shift protects the cells against subsequent menadione toxicity. These results are in line with recent publications that show a similar protective effect of the autophagic phenotype [54–56]. E.g. in tumor cells, degradation of the apoptotic initiator caspase 8 by autophagy was observed, resulting in reduced cellular stress and apoptosis. Likewise, in cholestasis, hepatotoxicity can be prevented by autophagy resulting in diminished ROS exposure. Autophagy is also involved in degradation of saturated fatty acids that induce hepatotoxicity [54–56]. Interestingly, in our study we demonstrate that the shift towards autophagy after oxidative stress induction is accompanied by increased degradation of Core protein in hepatocytes. Since Core is a more potent inducer of oxidative stress than NS3/4A, this would also explain the reduced oxidative stress in hepatocytes expressing Core (Figure 1) and it also explains the role of the antioxidant NAC in reversing the adaptive mechanisms in the model of several sources of damage. Additional experiments are necessary to confirm the role of autophagy and CMA in HCV Core degradation and the consequent resistance to apoptosis due to oxidative stress. Our results do not unequivocally demonstrate that autophagy was involved in Core degradation. However, the simultaneous degradation of p62/SQSTM1 supports the idea that autophagy-related proteins may be involved in degradation of stress factors like Core.

In summary, we demonstrate that expression of HCV proteins Core and NS3/4A induce a mild apoptotic and oxidative stress response in hepatocytes and this resistance attenuates the toxic effect of subsequent oxidative stress. The resistance to oxidative stress involves increased expression of protective anti-oxidant genes like HO-1, a shift towards an autophagic phenotype and a corresponding decrease in one of the stressors (Core protein) and reduced ER stress. Our study provides novel insights in the mechanism by which HCV infected hepatocytes adapt to survive in a hostile environment and suggests novel targets for intervention. Although we focused in our study mainly on HCV proteins Core and NS3/4A it will be interesting to evaluate additional HCV proteins like NS5 or even to extend these studies to other (hepatitis) viruses.

## Supplementary Material

Supplemental Material
