# Supplementary material for: Hepatitis C virus core or NS3/4A protein expression preconditions hepatocytes against oxidative stress and endoplasmic reticulum stress
Source: Redox Rep. 2019 Mar 25;24(1):17–26. doi: 10.1080/13510002.2019.1596431 (PMC6748607; doi:10.1080/13510002.2019.1596431)
Supplement: Supplemental Material [file YRER_A_1596431_SM9061.doc]

**SUPPLEMENTARY FIGURES**

**Supplementary Figure 1. Expression of HCV core and NS3/4A in Huh-7 cells.** (a) Graphic representation of the HCV constructs pTracerEmpty, pTracerCore and pTracerNS3/4A generated from HCV JFH1 strain. (b) Transfection efficiency was determined by FACS based on GFP expression of transfected cells at 30 hpt. A representative experiment (histogram) and the average of three independent experiments are shown. (c) Cell viability of transfected cells was determined using trypan blue exclusion staining. (d) The expression of HCV Core and NS3/4A was demonstrated by Western blot in Huh-7 cells 30 hpt using anti-HCV Core and anti-NS3/4A antibodies. The expression of GAPDH was used as loading control. (e) The expression of HCV Core and NS3/4A was also demonstrated by immunofluorescence in Huh-7 cells 30 hpt using anti-HCV Core and anti-NS3/4A antibodies, the viral proteins are indicated by white arrows. Huh-7 cells transfected with the empty vector were used as a control. Red arrows in (a) indicated the restriction sites. P_EF-1a_= Elongation factor 1a promotor. BGHpA= Bovine growth hormone polyadenylation signal. P_CMV_=Cytomegalovirus Promotor. SV40pA= Simian virus 40 polyadenylation signal.

**Supplementary Figure 2. Oxidative stress induction using menadione treatment.** Huh-7 cells were seeded in 6-well plates 24 h prior to Menadione treatment (50μM) for 6 h. Total ROS were quantified using CellROX fluorogenic probe according to manual instructions. As a control, Huh-7 cells were pre-treated with NAC during 30 min before oxidative stress induction. *t* test was performed to compare the means of the signal intensity and the asterisks represent *p* values: **<0.06 and *<0.02. (*p* value>0.05).

**Supplementary Figure 3. Transfection of rat primary hepatocytes.** (a) Transfection efficiency was determined by FACS based on GFP expression of transfected cells. A representative experiment (histogram) and the average of three independent experiments are shown. (b) The expression of HCV Core and NS3/4A was confirmed by Western blotting 24 hpt using anti-HCV Core and anti-NS3/4A antibodies. The expression of GAPDH was used as loading control.

**Supplementary Figure 4. Starvation of Huh-7 cells.** Huh-7 cells were seeded and 24 h were subjected to serum and glucose starvation for 2 h. Then cells were harvested and lysed. LC3-I/II and p62 were determined by western blot using specific antibodies. Densitometry analysis were performed to LC3-I/II and p62 protein expression. *t* test was performed to compare the means of the signal intensity and the asterisks represent *p* values: *<0.02. (*p* value>0.05). MW=Molecular weight.

**SUPPLEMENTARY TABLES**

**Supplementary Table 1. Primer sets for HCV Core and NS3/4A cloning.**

| **Name** | **Sense** | **Fragment*** | **Sequence (5´ - 3´)** |
| --- | --- | --- | --- |
| **Core_F** | Forward | 365 to 386 | GAGAGAATTCACCACCATGAGCACAAATCCTAAACCTT |
| **Core_R** | Reverse | 920 to 937 | GAGATCTAGAAGCAGAGACCGGAACGGT |
| **NS3/4A_F** | Forward | 3455 to 3473 | GAGAGAATTCACCACCATGGCTCCCATCACTGCTTATG |
| **NS3/4A_R** | Reverse | 5491 to 5509 | GAGATCTAGAGCATTCCTCCATCTCATCA |

*Fragment represent the nucleotide position for annealing, based on the full-length HCV JFH1 replicon genotype 2a, strain reference APP1025 (Apath)

**Supplementary Table 2.** Detailed description of the William´s E Medium, GlutaMAX™ Supplement (Gibco, Cat N 32551020).

| **Components** | **Concentration (mg/L)** | **mM** |
| --- | --- | --- |
| Glycine | 50.0 | 0.6666667 |
| L-Alanine | 90.0 | 1.011236 |
| L-Alanyl-L-Glutamine | 434.0 | 2.0 |
| L-Arginine | 50.0 | 0.28735632 |
| L-Asparagine-H2O | 20.0 | 0.13333334 |
| L-Aspartic acid | 30.0 | 0.22556391 |
| L-Cysteine | 40.0 | 0.3305785 |
| L-Cystine 2HCl | 26.07 | 0.08329073 |
| L-Glutamic Acid | 50.0 | 0.34013605 |
| L-Histidine | 15.0 | 0.09677419 |
| L-Isoleucine | 50.0 | 0.3816794 |
| L-Leucine | 75.0 | 0.57251906 |
| L-Lysine hydrochloride | 87.46 | 0.47792348 |
| L-Methionine | 15.0 | 0.10067114 |
| L-Phenylalanine | 25.0 | 0.15151516 |
| L-Proline | 30.0 | 0.26086956 |
| L-Serine | 10.0 | 0.0952381 |
| L-Threonine | 40.0 | 0.33613446 |
| L-Tryptophan | 10.0 | 0.04901961 |
| L-Tyrosine disodium salt dihydrate | 50.65 | 0.19406131 |
| L-Valine | 50.0 | 0.42735043 |
| Ascorbic Acid | 2.0 | 0.011363637 |
| Biotin | 0.5 | 0.0020491802 |
| Choline chloride | 1.5 | 0.010714286 |
| D-Calcium pantothenate | 1.0 | 0.002096436 |
| Ergocalciferol | 0.1 | 2.5188917E-4 |
| Folic Acid | 1.0 | 0.0022675737 |
| Menadione sodium bisulfate | 0.01 | 3.6231882E-5 |
| Niacinamide | 1.0 | 0.008196721 |
| Pyridoxal hydrochloride | 1.0 | 0.004901961 |
| Riboflavin | 0.1 | 2.6595744E-4 |
| Thiamine hydrochloride | 1.0 | 0.002967359 |
| Vitamin A (acetate) | 0.1 | 3.0487805E-4 |
| Vitamin B12 | 0.2 | 1.4760147E-4 |
| alpha Tocopherol phos. Na salt | 0.01 | 1.8027762E-5 |
| i-Inositol | 2.0 | 0.011111111 |
| Calcium Chloride (CaCl2) (anhyd.) | 200.0 | 1.8018018 |
| Cupric sulfate (CuSO4-5H2O) | 1.0E-4 | 3.9999998E-7 |
| Ferric sulfate (FeSO4-7H2O) | 1.0E-4 | 3.5971223E-7 |
| Magnesium Sulfate (MgSO4) (anhyd.) | 97.67 | 0.8139166 |
| Manganese Sulfate (MnSO4-H20) | 1.0E-4 | 5.9171595E-7 |
| Potassium Chloride (KCl) | 400.0 | 5.3333335 |
| Sodium Bicarbonate (NaHCO3) | 2200.0 | 26.190475 |
| Sodium Chloride (NaCl) | 6800.0 | 117.24138 |
| Sodium Phosphate monobasic (NaH2PO4) anhydrous | 140.0 | 1.0144928 |
| Zinc sulfate (ZnSO4-7H2O) | 2.0E-4 | 6.9444445E-7 |
| D-Glucose (Dextrose) | 2000.0 | 11.111111 |
| Glutathione (reduced) | 0.05 | 1.6286645E-4 |
| Methyl linoleate | 0.03 | 1.0169491E-4 |
| Phenol Red | 10.0 | 0.026567481 |
| Sodium Pyruvate | 25.0 | 0.22727273 |

**Supplementary Table 3.** Detailed description of the DMEM, high glucose, GlutaMAX™ Supplement, pyruvate Medium (Gibco, Cat N 10569010)

| **Components** | **Concentration (mg/L)** | **mM** |
| --- | --- | --- |
| Glycine | 30.0 | 0.4 |
| L-Alanyl-Glutamine | 862.0 | 3.9723501 |
| L-Arginine hydrochloride | 84.0 | 0.39810428 |
| L-Cystine 2HCl | 63.0 | 0.20127796 |
| L-Histidine hydrochloride-H2O | 42.0 | 0.2 |
| L-Isoleucine | 105.0 | 0.8015267 |
| L-Leucine | 105.0 | 0.8015267 |
| L-Lysine hydrochloride | 146.0 | 0.7978142 |
| L-Methionine | 30.0 | 0.20134228 |
| L-Phenylalanine | 66.0 | 0.4 |
| L-Serine | 42.0 | 0.4 |
| L-Threonine | 95.0 | 0.79831934 |
| L-Tryptophan | 16.0 | 0.078431375 |
| L-Tyrosine disodium salt dihydrate | 104.0 | 0.39846742 |
| L-Valine | 94.0 | 0.8034188 |
| Choline chloride | 4.0 | 0.028571429 |
| D-Calcium pantothenate | 4.0 | 0.008385744 |
| Folic Acid | 4.0 | 0.009070295 |
| Niacinamide | 4.0 | 0.032786883 |
| Pyridoxine hydrochloride | 4.0 | 0.019417476 |
| Riboflavin | 0.4 | 0.0010638298 |
| Thiamine hydrochloride | 4.0 | 0.011869436 |
| i-Inositol | 7.2 | 0.04 |
| Calcium Chloride (CaCl2) (anhyd.) | 200.0 | 1.8018018 |
| Ferric Nitrate (Fe(NO3)3"9H2O) | 0.1 | 2.4752476E-4 |
| Magnesium Sulfate (MgSO4) (anhyd.) | 97.67 | 0.8139166 |
| Potassium Chloride (KCl) | 400.0 | 5.3333335 |
| Sodium Bicarbonate (NaHCO3) | 3700.0 | 44.04762 |
| Sodium Chloride (NaCl) | 6400.0 | 110.344826 |
| Sodium Phosphate monobasic (NaH2PO4-H2O) | 125.0 | 0.9057971 |
| D-Glucose (Dextrose) | 4500.0 | 25.0 |
| Phenol Red | 15.0 | 0.039851222 |
| Sodium Pyruvate | 110.0 | 1.0 |

**Supplementary Table 4. Primer sets and probes for qPCR.**

| **Name** | **Type** | **Specie** | **Sequence (5´- 3´)** |
| --- | --- | --- | --- |
| **18S_F** | P. Forward | Human/Rat | CGG CTA CCA CAT CCA AGG A |
| **18S_R** | P. Reverse | Human/Rat | CCA ATT ACA GGG CCT CGA AA |
| **18S_P** | Probe | Human/Rat | CGC GCA AAT TAC CCA CTC CCG A |
| **HO-1_F** | P. Forward | Human | GAC TGC GTT CCT GCT CAA CAT |
| **HO-1_R** | P. Reverse | Human | GCT CTG GTC CTT GGT GTC ATG |
| **HO-1_P** | Probe | Human | TCA GCA GCT CCT GCA ACT CCT CAA AGA G |
| **MnSOD2_F** | P. Forward | Human/Rat | CAC CGA GGA GAA GTA CCA CGA |
| **MnSOD2_R** | P. Reverse | Human/Rat | GAA CTT CAG TGC AGG CTG AAG A |
| **MnSOD2_P** | Probe | Human/Rat | CCT GAG TTG TAA CAT CTC CCT TGG CCA G |
| **CuZnSOD_F** | P. Forward | Human | CTCACTTTAATCCTCTATCCAGAAAACA |
| **CuZnSOD_R** | P. Reverse | Human | ATCTTTGTCAGCAGTCACATTGC |
| **CuZnSOD_P** | Probe | Human | CAACATGCCTCTCTTCATCCTTTGGCC |
| **CAT­_F** | P. Forward | Human | TTC GAT CTC ACC AAG GTT TGG |
| **CAT­_R** | P. Reverse | Human | GTT GCT TGG GTC GAA GGC TAT |
| **CAT­_P** | Probe | Human | CAC AAG GAC TAC CCT CTC ATC CCA GTT GG |
| **GPx1_F** | P. Forward | Human | CGGCTTCCCGTGCAAC |
| **GPx1_R** | P. Reverse | Human | GAGGGAATTCAGAATCTCTTCGTTC |
| **GPx1_P** | Probe | Human | TGGCGTTCTCCTGATGCCCAAACT |
| **GRP78_F** | P. Forward | Human | TGG TGA TCA AGA TAC AGG TGA CCT |
| **GRP78_R** | P. Reverse | Human | GTG TTC CTT GGA ATC AGT TTG GT |
| **GRP78_P** | Probe | Human | TCC CCT TAC ACT TGG TAT TGA AAC TGT GGG |
| **sXBP1_F** | P. Forward | Human | GCT GAG TCC GCA GCA GGT |
| **sXBP1_R** | P. Reverse | Human | CCC AAA AGG ATA TCA GAC TCA GAA TC |
| **sXBP1_P** | Probe | Human | CCC AGT TGT CAC CTC CCC AGA ACA TCT |
| **ATF4_F** | P. Forward | Human | CAG CAA GGA GGA TGC CTT CT |
| **ATF4_R** | P. Reverse | Human | CCA ACA GGG CAT CCA AGT C |
| **ATF4_P** | Probe | Human | CCA TTT TCT CCA ACA TCC AAT CTG TCC C |
| **DDIT3_F** | P. Forward | Human | GGAAATGAAGAGGAAGAATCAAAAAT |
| **DDIT3_R** | P. Reverse | Human | GTTCTGGCTCCTCCTCAGTCA |
| **DDIT3_P** | Probe | Human | TTCACCACTCTTGACCCTGCTTCTCTGG |
| **HO-1_F** | P. Forward | Rat | TGGCGTTCTCCTGATGCCCAAACT |
| **HO-1_R** | P. Reverse | Rat | CTG GTC TTT GTG TTC CTC TGT CAG |
| **HO-1_P** | Probe | Rat | CAG CTC CTC AAA CAG CTC AAT GTT GAG C |
| **CuZnSOD_F** | P. Forward | Rat | CCGTACAATGGTGGTCCATGA |
| **CuZnSOD_R** | P. Reverse | Rat | CCCAGCATTTCCAGTCTTTGTACT |
| **CuZnSOD_P** | Probe | Rat | CTT CAT TTC CAC CTT TGC CCA AGT CAT C |
